# “Layers of translation” - evidence literacy in public health practice: a qualitative secondary analysis

**DOI:** 10.1186/s12889-017-4837-z

**Published:** 2017-10-11

**Authors:** Wanda Martin, Joan Wharf Higgins, Bernadette (Bernie) Pauly, Marjorie MacDonald

**Affiliations:** 1 College of Nursing, University of Saskatchewan, PO Box 6, 104 Clinic Place, Saskatoon, SK S7N 2Z4 Canada; 2 Exercise Science, Physical & Health Education, University of Victoria, Box 1700 STN CSC, Victoria, BC V8W 2Y2 Canada; 3 School of Nursing, Centre for Addictions Research of BC, University of Victoria, Box 1700 STN CSC, Victoria, BC V8W 2Y2 Canada

**Keywords:** Evidence literacy, Secondary analysis, Evidence-based, Evidence-informed

## Abstract

**Background:**

Strengthening public health systems has been a concern in Canada in the wake of public health emergencies. In one Canadian province, British Columbia, a high priority has been placed on the role of evidence to guide decision making; however, there are numerous challenges to using evidence in practice. The National Collaborating Centre for Methods and Tools therefore developed the Evidence Informed Public Health Framework (EIPH), a seven step guide to assist public health practitioners to use evidence in practice. We used this framework to examine the evidence literacy of public health practitioners in BC.

**Methods:**

We conducted a secondary analysis of two separate qualitative studies on the public health renewal process in which the use and understanding of evidence were key interview questions. Using constant comparative analysis, we analyzed the evidence-related data, mapping it to the categories of the EIPH framework.

**Results:**

Participants require both data and evidence for multiple purposes in their daily work; data may be more important to them than research evidence. They are keen to provide evidence-based programs in which research evidence is balanced with community knowledge and local data. Practitioners recognise appraisal as an important step in using evidence, but the type of evidence most often used in daily practice does not easily lend itself to established methods for appraising research evidence. In the synthesis stage of the EIPH process, synthesized evidence in the form of systematic reviews and practice guidelines is emphasized. Participants, however, need to synthesize across the multiple forms of evidence they use and see the need for more skill and resources to help them develop skill in this type of synthesis.

**Conclusions:**

Public health practitioners demonstrated a good level of evidence literacy, particularly at the collective level in the organization. The EIPH framework provides helpful guidance in how to use research evidence in practice, but it lacks support on appraising and synthesizing across the various types of evidence that practitioners consider essential in their practice. We can better support practitioners by appreciating the range of evidence they use and value and by creating tools that help them to do this.

## Background

Early in the twenty-first century, many people raised concerns in Canada about the Public Health (PH) system’s capacity to meet growing population health needs and address PH emergencies (e.g. pandemics) [1, 2]. This stimulated PH renewal policies and processes across the country. A component of the plan to strengthen PH in one Canadian province, British Columbia (BC), was the development and implementation of a *Framework for Core Functions in Public Health* [3]. This specified the PH services and supports that the BC Ministry of Health (MoH) expected regional health authorities to provide to their communities [4]. A description of the framework has been published elsewhere [5, 6]. In implementing this PH renewal process, the Ministry gave high priority to the role of evidence to guide decision making.

The importance of evidence-informed policy and practice [7, 8] is acknowledged in PH [9–12] yet an evidence to practice gap remains [13, 14]. Challenges to using evidence in PH practice include: 1) finding time in an already challenging workload; 2) politicized decision-making for which the best evidence may not be considered for political reasons; 3) lack of resources when the best approach may be considered too expensive; 4) organizational culture and other factors involving lack of leadership, limited support for research, and limited skills, knowledge, and critical appraisal tools [15, 16].

### Conceptual framework for the study

To support Canadian PH practitioners and policy makers in using evidence to inform practice, six National Collaborating Centres for PH were developed to “promote and improve the use of scientific research and other knowledge to strengthen PH practices and policies in Canada” [17]. Each centre develops knowledge translation tools relevant to the focus of that centre. One of these centres, the National Collaborating Centre for Methods and Tools (NCCMT), has developed an “Evidence Informed PH Framework” (EIPH) [18] to guide PH practitioners to find, distill and use high quality evidence. PH practitioners include public health and preventive medicine residents and doctors, public health nurses, community nutritionists, health inspectors, and others who perform public health functions. The framework is well supported by a highly accessed registry of methods and tools to support evidence informed public health [19]. The framework states that evidence-informed decision making in PH takes into account evidence from: 1) the community and local context; 2) community and political preferences and actions; 3) PH resources; 4) research; and 5) PH expertise. It provides a set of seven steps to guide the process (see right hand column in Table 1).

**Table 1:** Comparison of literacy frameworks

Steps in literacy frameworks		
Health literacy	Information literacy	Evidence-informed public health
–	Clarifying the Problem	Define the problem
Access health information	Locating Sources	Search
Understand	Selecting/Analyzing	–
Evaluate	–	Appraise
–	Organizing/Synthesizing	Synthesize/Interpret
–	–	Adapt
Use	–	Implement
Communicate	Creating/Presenting	–
–	Evaluating	Evaluate

We noted significant parallels between these steps and those of information literacy and health literacy frameworks. In Table 1 we compare the steps in the processes of developing health literacy [20, 21], information literacy [22, 23], and EIPH [7, 10]. Inspired by these similarities among frameworks for using information, we thought the EIPH framework would be appropriate to guide our analysis of evidence-related data in PH practice to determine the extent of evidence literacy among PH practitioners in BC. Furthermore, a study evaluating the utility of tools to guide each step in the EIPH framework was previously published in this journal [7]. We defined evidence literacy as *the ability to: define the PH problem at hand and the need for evidence; search for and locate relevant evidence; appraise, interpret, adapt, and implement the appropriate evidence; and evaluate the use of evidence to inform public health programs.*

### Study purpose

In the context of a large program of research examining the renewal of PH systems in British Columbia [5, 6], specifically the implementation and impact of the Core Public Health Functions Framework [3], two studies were initiated, the Healthy Living (HL) study and the Knowledge-to-Action (KTA) study. The background on the HL and KTA studies is provided in Table 2. In these studies, the primary investigators (who include the authors of this paper) explored the evidence-to-practice processes in three health authorities and three core PH programs in BC. In this paper, we present the findings of a secondary analysis of these data. The first primary study focussed on the healthy living core program in Health Authorities (HAs) A and B, while the second examined the food safety and unintentional injury prevention core programs in HAs A, B, and C.

**Table 2:** Description of primary studies

Studies	Healthy living	Knowledge to action
Purpose	To explore the implementation process for the HL core public health program, including the context within which practitioners implemented this program, and the factors influencing implementation – in particular, how practitioners used evidence in implementation decisions.	To explore the knowledge-to-action process and to identify and assess appropriate knowledge translation strategies to support the use of evidence in core program development and implementation, and to compare the process and outcomes across health authorities.
Data collection	29 individual interviews	49 individual interviews and 3 focus groups
Participants	HA 1 (*n* = 14), HA 2 (*n* = 7) and MoH (*n* = 8). Public health managers (*n* = 9) and front line staff (*n* = 12) from urban and rural areas in two health authorities	HA 1 (*n *= 13), HA 2 (*n* = 13), and HA 3 (*n* = 16), BC Centre for Disease Control (a provincial level organization, *n* = 1), the BC Injury Research and Prevention Unit (*n* = 3), and the MoH (*n* = 3) Individual interviews (*n* = 10) and 3 focus groups (n = 9) with front line food safety staff and managers and individual interviews (*n* = 30) with injury prevention staff and managers
Interviewers	Two of the principal investigators (one being author Wharf-Higgins) and the research coordinator	The project coordinator (author Martin) and three trained research assistants

The research question for this secondary analysis was: To what extent are PH practitioners who work in these three program areas evidence-literate? Using the EIPH Framework as a guide for the analysis, we assessed practitioners’ evidence literacy. We also assessed the utility of the EIPH framework for guiding PH practitioners in evidence-informed decision making.

## Methods

### Secondary analysis

There is a long history of secondary analysis in quantitative research, but it is relatively new in qualitative research, although increasingly more common [24–26]. “Secondary analysis involves the utilisation of existing data, collected for the purposes of a prior study, in order to pursue a research interest which is distinct from that of the original work” (paragraph 1) [27]. It may involve using data from single or multiple data sets shared by the primary investigators formally (e.g., in a data archive) or informally with other researchers, or it may involve the use of existing data gathered in primary research by the same researchers conducting the secondary analysis [28].

In this study, researchers for the primary studies were involved, and shared two data sets for analysis. Ethical approval for secondary analysis was included in the original ethics application for the KTA study (HREB#s: UVic/VIHA J2008–41; VCH CS08–040; IH 2008–050). A modification of the original ethics application to the appropriate ethics boards was approved for secondary analysis of the HL data (HREB# BC15–364). Both studies received ethical approval from the University of Victoria/Vancouver Island Health Authority Joint Research Ethics Board and from Vancouver Coastal Health Authority. The KTA study also had approval from Interior Health Authority.

### Data analysis

Interviews from the KTA study were entered into NVIVO 10 software. The evidence-related data in the KTA data set were originally coded into 14 categories or nodes (see Table 3). We reviewed these data for relevance to the new research question. Finding it appropriate, we retained these 14 codes as the starting place for the secondary analysis.

**Table 3:** NVIVO nodes

1. How practitioners understood and defined evidence	
2. Challenges associated with using evidence	
3. Changes that occurred as a result of using evidence	
4. How evidence guides or does not guide practice	
5. Debates about the evidence	
6. Role of the model core public health program paper in developing a performance improvement plan	
7. The purpose for which evidence is needed	
8. Traditional ways of working with respect to evidence use	
9. Types and sources of evidence used	
10. Uptake of the provincial public health program evidence reviews	
11. Ways of thinking about evidence in general	
12. The concept of evidence-informed practice	
13. The concept of practice-informed evidence	
14. Ways of incorporating various knowledge types into practice	

Merging the HL data set with the KTA data set, the first author (WM) coded all the HL study evidence-related data into the 14 KTA nodes. It was unnecessary to create additional nodes for the HL data because the original KTA nodes were sufficient to accommodate the HL data and to answer the new research question. As a group, we then reviewed and recoded the merged data within each of the 14 parent nodes into new nodes based on the seven steps in the EIPH framework: define, search, appraise, synthesize/interpret, adapt, implement and evaluate. We created sub-categories (or child and grandchild nodes) to analyze the dimensions and processes inherent in each of the higher level parent nodes, grouping and regrouping codes at higher levels of abstraction. Three of the investigators (JWH, WM, MM) divided the steps among them and each coded and analyzed the data for steps assigned to them. All four investigators reviewed, critiqued, and discussed the analysis for each step, editing as necessary until consensus on the analysis and conclusions were reached.

## Results

To understand the extent to which PH practitioners are ‘evidence-literate’, it is helpful to know how research participants understand the term evidence and what evidence means to them. Therefore, we begin this section with a discussion of participant understandings and definitions of evidence.

### How is the term evidence understood or defined?

*“I don’t know if I have a specific definition for ‘evidence’, but I think there’s a broad range of different types of evidence that have different strengths and weaknesses.”*

In discussing what they believe counts as evidence, participants identified a wide range of evidence types and sources including: research, systematic reviews, literature reviews, surveys, surveillance data, population health data, coroner’s data, program evaluation, expert opinion, experience, epidemiological data, community-based data, and anecdotal evidence from colleagues and community members.

Overall, participants believed that evidence was important to inform PH practice and most reported using many types of evidence in diverse ways. Largely, they held very broad understandings of the term evidence. Although participants recognized that scientific evidence from research is important to consider, many also valued community-based knowledge very highly. Most saw the need to bring both types of evidence together recognizing that the scientific evidence needed to be contextualized for and relevant to the community.

“And I think we have to [define evidence broadly]. Because you can’t take what works, statistically, with this group and transform it to this group of citizens living in a low income housing unit, for example. Right? Like, you have to have some kind of needs assessment evidence from the community and then try to find some sort of hybrid as to what the best practice says” (Study Participant).

Some participants, particularly medical health officers, tended to value epidemiological data and research-derived evidence more highly. On the other hand, those working in food security preferred to draw on community-based experiential evidence over the scientific evidence, seeing it as more valid.

But evidence for me is community-based evidence that I see every day. So, the food security work that I do is so grass roots that the evidence just comes from the truth of what I’m being told by the people in my community.

Although many participants view evidence as important in guiding and informing their practice, they also believe that evidence is not the only thing to take into consideration. As one person described, it is not just about evidence, but “it’s the process and sometimes it can come back and bite us,” suggesting that lived experience and community development processes are important considerations in public health decision-making. Although evidence, in whatever form, is important to consider as a resource, it is not necessarily the main driver of the work.

### Findings related to the steps in the EIPH framework

#### Step 1 - defining the question

*“When we start seeing a need come out, often times it’s, you know, we’re approached by clients or a group of clients that have come together and said, “You know, we’re seeing a problem with x and such and such. Can you please help us out with it?””*

The first step in the EIPH framework is defining the question. This step answers the questions: *“Who is my target group? What is the issue we are dealing with? And what specifically are we trying to change?”* [18]. Participants did not talk specifically about target groups, in part because this was assumed or evident in the work they were doing. They did, however, discuss the issue they were dealing with in their comments about the reasons they needed evidence. In the quotation above, one participant noted that the issue was identified by the community in request for help in addressing it, thus creating a community-driven need for evidence. Other participants not only wanted evidence about what works to produce desired outcomes, but also wanted local epidemiological data to define the scope and extent of a PH problem that could be used to: 1) achieve specific aims or goals; 2) inform PH processes; 3) help set priorities; 4) justify a new program or policy; 5) assess changes over time as a result of an intervention; 6) monitor or track population health status or health trends; or 7) justify expenditures.

Each participant spoke about the specific health issues on which they were working, such as food safety, food security, various injuries or the causes of injuries such as falls or traffic crashes. However, they talked less about needing evidence on a specific health issue and more about needing evidence to inform processes or practices in the work they were doing. Many participants wanted evidence to demonstrate that an issue was indeed a problem that needed to be addressed more than they wanted evidence about the effectiveness of a program they might be considering. Similarly, others were interested in evidence to justify programs or policies already implemented rather than evidence of effective programs, as reflected in the following quotation:

“So there isn’t a lot of traction in going down the road with this, so anything I can get that has some weight to it, including some research backing, whether it is specific to brain injury or not, but something that says this is an area of practice that matters to the health regions gives me justification for staying involved” (Study Participant).

Several participants also expressed a need for data or evidence to document the work they were doing, what that meant, and whether it was effective. This could include locally relevant process data or evaluation evidence on the effectiveness of their work. Demonstrating that their work was having an impact was important because, with all the organizational changes and budget cuts going on in the health authorities, participants wanted to demonstrate the value of their work in hopes that additional resources could be obtained. As one participant stated: “We only have two dietitians for the whole island so it would be great to be able to show that what we’re doing is effective and we need more of it.”

Each participant identified specific changes they wanted to document through evidence such as: improving population health, reducing the burden of injury, improving indigenous health, developing safety standards, and shifting the health authority mindset from treatment to prevention. For these things, they believe they need locally generated epidemiological and evaluation data as well as research evidence of effectiveness.

#### Step 2 – Searching for evidence

*“It’s so much easier to Google these things now: has anywhere else in the world done this?”*

This second step in the EIPH framework asks the question *“Where should I look to find the best available research evidence to address the issue?*” [18]. The question itself implies that research evidence is the most important information for which practitioners should be searching. As discussed above, participants talked about searching for different kinds of data to guide their work, not just research evidence.

With respect to helpful sources for locating evidence, participants identified provincial and national sources, professional groups, disease-based foundations, Health Authorities, or local groups and networks as important. They were resourceful and creative in finding relevant and available sources of data. Some relied less on academic journals and more on grey literature, in part because grey literature is more accessible and understandable to them. Again, we note that participants equate data with evidence, even though it may not come from research.

Participants identified three sources of data for obtaining the best available evidence: formal research, big data, and practice. In *formal research*, participants said it was important to have access to a university library to track down references and not all of them had that access. They might search for systematic reviews from the Cochrane Database, or other research sites. Randomized controlled trials were important to some, but seen as not always feasible in PH. Expert organizations (e.g., WHO) were important sources because they are trusted groups that provide good evidence summaries. When it comes to searching for research, however, practitioners often relied on others to do this work:

“Personally, I usually rely on others. I look to my colleagues in Population Health and Wellness. They are usually the ones who have pulled together the lit searches. They have some program experts, so I go to them assuming that I am going to get the latest and greatest information” (Study Participant).

Most participants relied on electronic sources such as webcasts, list-serves, and e-mail lists to get updates on research. Distribution lists delivering monthly updates make it easy to scan for a match to something they might be working on. Connecting with peers and colleagues to ask for information is a common practice; finding out what others are doing is easier now with Google.

For some participants, searching for evidence meant looking to *big data* sets like the Canadian Community Health Survey or to medical service plan and hospital data. But, not everyone has the skills or confidence to access these data sources; having others available who can do this is important. At the same time that access to data was important, one participant cautioned about hiding behind the need for data before moving to action:

“I think we often hide behind getting the data. We always tell ourselves, we need more data, we need more data. It needs to be more applicable. It needs to be more localized. Well we can move forward still. You don’t have to be stymied by waiting for data. I think sometimes we’re always looking for evidence” (Study Participant).

This participant recognizes that there is often a tension between the need for data and the need for action. If local data are not available, waiting for it may delay action unnecessarily, hence *big data* might be better than no data. For a local perspective, however, practice can be a valuable source of information.

For many participants, searching for evidence largely occurred within practice. This involved looking in-house to clinical records, to the strategic information department, vital statistics, or various medical health officers’ reports that identify local priorities and information. They also commented on evidence being embedded in policies, guidelines and regulations found within the context of their work. As discussed previously, many participants displayed a strong preference for obtaining evidence from their communities. Having hands-on experience and getting community input was important evidence for them. They found evidence through windshield surveys or needs assessments. Thus, the community voice is an important part of searching for evidence, and seen by many as the ‘truth’ or the most valid form of evidence.

In some cases, locating evidence was informal through sharing at conferences, or talking with local experts, colleagues, and networks, or through partnerships with Masters student projects. In a few instances, evidence-sharing involved formal relationships with researchers. Despite the variety of sources accessed by participants, some identified particular challenges when searching for evidence. For example, access to data might be restricted by organizational agreements or privacy regulations. Practitioners often see too much formalization related to data access.

#### Step 3 – Appraising evidence

*“Well the qualifier then is, what is the quality of that evidence?”*

The third step in the EIPH framework is critical appraisal. Here, the quality of study methods is assessed to ascertain whether its findings are “trustworthy, meaningful and relevant” for uptake [18]. The question asked in this step is: “*Were the methods used in this study good enough that I can be confident in the findings?”* Participants clearly recognized the importance of critical appraisal skills for their work:

“But I certainly think that we need to be cognizant of the quality of evidence that we’ve got and what are the shortcomings and strengths of the type of evidence that we have. And where evidence is weak, i.e., it’s not coming from a gold standard type of methodology” (Study Participant).

Although some articulated a preference for a traditional hierarchy of research designs, this was tempered by the realization that such studies were not always feasible or ethical to conduct in PH and that other types of research could be useful.

“So I guess while, you know, random controlled studies are seen as the best evidence, the ultimate, I’m not sure that they’re the only sources of evidence. Certainly, we do have to look at those other sources as well” (Study Participant).

Some interviewees spoke about “relenting” to the idea of accepting “promising practices” as evidence in PH when higher quality evidence was not available. At the same time, they recognized the need to be critical about the evidence: “So hopefully being a little bit more critical about what we take in and look at and use. Not just taking it and going ‘well, this sounds good.’ Plop. Let’s use it.” On the other hand, as discussed above in the *defining the question* and *searching for evidence* sections, several participants preferred evidence that came from the community, and their confidence in the evidence relates to how well the evidence is connected to the local context.

Reports of local projects or programs may not provide strong evidence of success but may be “good enough” to adapt in the local context and then evaluate, thus contributing to the evidence base. But what constitutes “good enough”? Practitioners have little guidance on making these decisions and the EIPH appraisal step focusses only on appraising research evidence. There may be a risk in taking up an unstudied intervention, but it may be all that is available. Coupled with evaluation, however, participants believe that it can provide useful information.

Although not related to the specific appraisal question in the EIPH framework, our participants also spelled out the characteristics of evidence that are important for PH practitioners. They appraise the usefulness of evidence on the extent to which it reflects these characteristics. In general, they require evidence (or data) that is timely, relevant to their context and purpose, current and regularly updated, synthesized and translated into manageable bite sized pieces, trustworthy, and of different types at different levels. Examples are presented in Table 4. In general, practitioners recognise appraisal as an important step in using evidence, but the type of evidence most often used in PH practice does not easily lend itself to methods for appraising research evidence.

**Table 4:** Characteristics of evidence for PH

	Quotes
Timely.	We want data to be timely. We want to know what’s going on now, not data that’s three years old and that’s always a hurdle for people to get over and get through and work through.
Relevant.	Canadian Dietitian’s Association has lots of diet evidence and it’s rated, but it’s mostly clinical right now. It’s starting to look at public health nutrition evidence and community nutrition practice [which is more relevant].
Regularly updated.	…to pull together the data to you know, update it on an ongoing basis to put it in front of the decision-makers.
Synthesized.	So being the age that I am and being very comfortable with Internet research, I often go to the Cochrane database first of all to have a look to see if there’s any evidence out there that people have agreed on.
At different levels.	Well the level of evidence – and we can go back to the old Canadian primary prevention guidelines. What were those called? The levels of evidence were a, b, c, d, and e.
Manageable bite sized pieces.	Unfortunately, it can’t be an evidence paper that’s ten pages long. It has to be something that at the operational level you can scan it and get the evidence bites out of it and then incorporate it into your plans.
Trustworthy.	If the CDC publishes something or the Ontario Tobacco Research Unit – we feel that it meets a certain standard that we can expect…. and because it’s from a stakeholder we trust, we read that evidence with interest.

#### Step 4 – Synthesizing and interpreting evidence

*“And you know, one needs to wonder… to what extent then after all those layers of translation, does the program that’s put on the ground or the quality improvement agenda resemble the type of evidence in the literature, and to what extent would that even be suited to the context.”*

In the fourth step of the EIPH framework, users are asked to interpret the evidence they have gathered in synthesized form to produce ‘actionable messages’. They are advised to produce recommendations from the “highest quality and most synthesized research evidence available” [18]. The question answered in this step of the EIPH is *“What does the research evidence tell me about the issue?”* [18]. The synthesis step in the EIPH does not focus on the synthesis process itself but rather on interpreting the evidence derived from already synthesized research. There is no discussion of how to synthesize the other types of evidence in the EIPH Model (i.e., community health issues and local context, community and political preferences and actions, PH resources, and PH expertise).

As intended in the EIPH framework, some participants report drawing on findings from systematic reviews and RCTs and interpreting this evidence to make decisions about practice:

“So if we’re trying to recommend, for example, the use of child restraints for motor vehicle safety, I mean we wouldn’t put out that recommendation as is. We would justify it and back it up with a lot of studies that have, you know, shown the use of this, that have been evaluated. We look at systematic reviews, we look at if at all there have been any recognized control trials about data showing the actual use of it” (Study Participant).

In addition to systematic reviews, some participants draw heavily on synthesis work as reflected in policy and position papers conducted by trusted agencies and organizations. Other participants discussed using clinical practice guidelines as exemplifying high quality evidence and embodying the synthesis process. This is very much in keeping with the evidence hierarchy discussed on the EIPH website.

“So these are the ‘treating tobacco use and dependence’ clinical practice guidelines, the 2008 update. So we use these as kind of like our bible for tobacco control. And that is probably what I would look at as the best evidence right now” (Study Participant).

What is very telling, however, is that many participants make reference to a synthesis and interpretive process that involves pulling together evidence from multiple sources such as community needs, local context, and practice experience, in addition to research. One participated stated:

“I think a lot of where we find our need is from the community partnerships that we have built with vulnerable populations and the different agencies that work with them. And then we kind of take that information and combine it with evidence that we have, and try to move forward with some kind of an initiative. So there is a real grassroots component to our evidence-finding, as well as taking academic and clinical knowledge and all of that, to make it work.”

Here, it is not just high quality research evidence that is being synthesized to make decisions about action but other types of knowledge are integrated with the research evidence. Consistent with the stated purpose of the EIPH that different types of evidence need to be considered in decision making, many participants are putting various types of information together, interpreting that data, and drawing implications for practice. The EIPH synthesis step, however, focuses primarily on using synthesized research evidence.

The lack of skills and capacity in the organization to do the synthesis work was a common theme in the data. In some cases, however, practitioners could rely on someone else, either a trusted organization or an in-house staff member who had the skills or for whom it was part of their job, as it was for the participant who made this comment: “It’s part of my job to keep up with the evidence that is there and then to incorporate that into all of the work that I do.” There is very little in the research literature, however, about the process of synthesizing other forms of evidence with research evidence. Participants expressed the need for support in doing this work.

#### Step 5 – Adapting evidence to the local context

*“It’s always a matter of adapting. It’s never straightforward – something that we can take from somewhere else and plunk it down here.”*

The fifth step of the EIPH framework is adapting the evidence to the local context. It addresses the question *“Can I use this research with my client, community, or population?”* [18]. Adapting the evidence is an important step when using it in practice, as stated by this participant: “There is lots of evidence but it doesn’t always really fit with what you’re doing”.

Several participants who talked about adapting the evidence were well grounded in their communities and expressed a strong sense of the importance of working with community members, not only in adapting evidence to apply to the community, but also in recognizing that initiatives coming from within the community should be made available as evidence for others. As one participant noted:

“I think we always adapt the guidance or evidence to our own community. And, it’s kind of always a partnership with other community groups or organizations that we’re working with and how does implementation make sense for our own community? Also, a lot of great initiatives have come from our own community that have gone or may be able to go and move elsewhere as well.”

The idea that “knowledge is in the communities” is important to consider when there is a strong focus on evidence-informed practice because participants believe that the local context influences program implementation and success. That is, local knowledge and community engagement can help to modify evidence-informed programs to make them fit the context. At the same time, as the participant quoted above stated “It’s never straightforward”.

#### Step 6 – Implementing evidence

*“I think, for the most part, if there is evidence, I think that would inform most of the planning, implementation, and evaluation of my work. Otherwise, you’re just kind of flying by the seat of your pants.”*

Implementing, or applying evidence, is the sixth step of the EIPH framework. It addresses the question “*How will I use the research evidence in my practice?”* [18]. At this stage, practitioners act on the evidence to make a practice change. Many participants reported relying on evidence to broadly inform their strategic operations, front-line planning, and the delivery of PH programs. They also used evidence to make funding and resource decisions, and to inform the adoption of indicators/benchmarks for evaluating their work.

“As we were doing program planning and implementation, we were basing it on sort of the best knowledge that we could get at that point in time. So, we initiated a number of new programs or new directives and we did try to collect the evidence before we did that to help us decide. So, for example, one was looking at postpartum depression. We then created a program plan for implementation” (Study Participant).

Some participants also talked about using evidence as part of communications: “As our Senior Medical Health Officer said in a conversation with me, like we’ve got to use the data to tell compelling stories.” Some expressed concern, however, that evidence might be cherry picked to reaffirm existing practices rather than to inform necessary policy or program changes:

“Because they would say that they look at the evidence and they probably do, but I think it’s used more in a symbolic use rather than an instrumental use. …. But symbolic use is more like, I’ve already made up my decision. I’m going to go and find some research to support my decision and I think that’s the strongest use of research in the Health Authority when it comes to decision-making” (Study Participant).

Thus, many participants report using evidence to inform program planning, implementation, funding and evaluation decisions and they say they try to use the best available evidence. At the same time, they also indicate that evidence may be used inappropriately in some circumstances to justify what is already being done rather than to make necessary changes.

#### Step 7 – Evaluating evidence use

*“So, we would use that form of evidence or research to check back on is this working kind of thing – benchmarks.”*

The seventh and final step in the EIPH framework is to evaluate the use of the evidence to inform PH programs. The questions addressed in this step are: “*Did we do what we planned to do?” “Did we achieve what we expected?”* [18]. The first is an implementation question; the second is an outcome question. Both are important and can be addressed through evaluation, which can contribute to the evidence base. Participants consistently agreed on the value of evaluation, specifying:

“I’ve thought of indicators for success, and that’s where evaluation comes in, of the programs that we are doing. And for the education piece, we did a formal evaluation of that through the research centre here, and we had time to do that”.

Additionally, participants recognized the challenges inherent in the process. Evaluation takes time, preparation, and knowledge. As one person noted:

“I think that sometimes I feel that I don’t necessarily have the time to do, sort of like a logic model: ‘this is what I’m going to do, these are the indicators, and that …’. You are just going so quickly, and trying to keep up with everything. We have minimal staff time, where you have a lot of things going on.”

Participants acknowledge that they may need help doing an evaluation, perhaps from a research centre in the organization. But time for evaluation is always limited. It seems that consistent evaluation, while recognized as valuable, is not factored into the regular workload and is not a high priority in the organization. A lack of funding may contribute to a tension between a recognized need for evaluation evidence to guide practice and the competition for scarce resources.

Another issue that influences whether evaluation is done is the organizational capacity for evaluation. Although some participants are comfortable with the evidence-informed process and see evaluation as a natural part of this, others are not as comfortable. Some have taken courses in evaluation and can provide some of that capacity, but overall, evaluation capacity is limited, and is not well supported in many organizations.

Some participants reported doing evaluation in a more informal and less rigorous way by asking simple questions like: “Have you learned anything from this program?” For many, a more informal process is all that is feasible for them with such limited resources available for evaluation.

“I think it’s hard to think of doing that in a formal process because we are so stretched thin that I don’t have time to come back and report that or make up a document saying this garden was successful because we had five people there digging today. You know, so it’s hard” (Study Participant).

Overall, evaluating the use of evidence or the success of evidence-informed programs is not at top of mind for participants, but they did identify the value of evaluating programs, recognizing the need for assistance and organizational capacity. They appear to rely more on informal evaluations to provide feedback for improvements.

## Discussion

Based on our findings, we will answer four questions in the discussion that follows: *How do PH practitioners understand and use evidence? How ‘literate’ are PH practitioners in using evidence to inform decision making? What can we do to improve evidence literacy? Do we need any adaptations/modifications to the EIPH framework or does it provide a helpful guide for evidence informed decision-making?* In addressing these questions, we draw on related literature and highlight the implications or recommendations for strengthening evidence literacy in PH with a focus on the EIPH framework.

### How do public health practitioners understand and use evidence?

A secondary analysis of two data sets, focused on the implementation of different PH programs, provided an opportunity to examine the meaning and use of evidence in PH practice using an evidence-informed public health framework. Participants in all three core PH programs across different health authorities described their broad understandings of the term “evidence” as well as their own use of what they considered the best available evidence [29]. They require evidence for many different purposes relying on more than one source or type of evidence, including research evidence; tacit knowledge from their professional experiences, expertise and observations; community judgments; theory; and the “cumulative wisdom derived from systematic analysis of these and an understanding of the situations and populations in which they would be applied” [30] (p. 127). In many instances, participants described using expert opinion and community perspectives – data that were culturally embedded and user defined [31] – to inform their work, often valuing this over research evidence. According to Chen [32] this establishes the ‘viable validity’ of evidence that is not always derived from research but is “practical, affordable, suitable … and helpful in the real world” (p. 207), a “paradigm-shifting” approach [33].

The experience of PH practitioners’ use and understanding of evidence has been explored in other health care systems including Australia [34], the United States [10], and the United Kingdom [35]. Klinner et al. [34] described the use of research and relationship-based approaches in evidence-informed health promotion practice. Similar to our findings, these authors identified the importance of knowledge generated through community development and relational processes as part of evidence informed practice. Ammerman et al. [13] noted that PH practitioners came to recognize the value of practice-based evidence long before the research community accepted this. Mirroring the findings of others [10, 36], participants in this study reiterated the importance of drawing on multiple sources and types of evidence that serve their pragmatic needs.

As in our study, Li et al. (2015) found that PH practitioners used evidence for both strategic and practical purposes. Overall, practitioners valued a combination of both “scientistic” (high-quality research-based evidence), and humanistic evidence that was generated by practitioners or communities. Humanistic evidence was valued for its practical implications and was perceived as more useful for guiding practice. Scientistic evidence was valued for strategic purposes, but was valued less highly. This is very consistent with the findings of our study. These understanding need to be better reflected in existing evidence-informed decision making frameworks such as the EIPH.

### How literate are public health practitioners?

In recognition that the steps in the EIPH framework mirror the steps in health and information literacy frameworks (see Table 1), we used this framework to assess qualitatively the extent of evidence literacy among PH practitioners in BC. To reiterate, our definition of health literacy is: “*the ability to: define the PH problem at hand and the need for evidence; search for and locate relevant evidence; appraise, interpret, adapt, and implement the appropriate evidence; and evaluate the use of evidence to inform public health practice.”*

#### Step 1: Defining the issue

PH practitioners draw on multiple sources of evidence – some participants privileging community knowledge and some privileging research knowledge but most recognizing the importance of many types of evidence, broadly defined. This is congruent with the premise of the EIPH framework that evidence from five sources are important to consider in evidence informed decision making in public health: the community and local context, community and political preferences / actions, PH resources, and PH expertise and research. Practitioners have pragmatic information needs that can best be addressed by many forms of evidence (or data). They use a range of evidence to help them define important PH issues.

#### Step 2: Searching for evidence

Participants were resourceful in their searches for evidence drawing on diverse sources, congruent with their broad definitions of evidence. They knew who to approach for assistance in their own organizations, which organizations could provide reliable and trustworthy evidence in a digestible form, and how to access information and evidence online. They were committed to using evidence, of various types, to inform their practice.

#### Step 3: Appraising evidence

Public health practitioners recognize the importance of appraising the research evidence for quality but observed that the type of evidence most often used in PH practice does not easily lend itself to methods for appraising research evidence. They have a more pragmatic set of criteria for appraising evidence than is outlined in the EIPH. Their criteria are based on: a) a view that multiple forms of evidence are needed and should be valued at least as highly as research evidence; b) how practical it is for use in planning and implementing programs; and c) how well it relates to the local context. They do not shy away from making judgements about evidence quality, but struggle with how to appraise the various forms of evidence to make decisions. The EIPH framework focusses only on appraisal of research evidence.

#### Step 4: Synthesizing and interpreting

Our participants use already synthesized evidence such as systematic reviews and clinical practice guidelines, but these are often not available. When necessary, they do engage in a synthesis process to integrate different types of evidence, but it is not a synthesis of research evidence and does not follow the process laid out in the EIPH framework. They experience challenges in putting all types of evidence together and would like guidance on how to do this better.

#### Step 5: Adapting evidence

The participants in our study state that they “always” adapt the evidence to the community context. The need for adaptation is absolutely central to their views of evidence, no matter the source, but they also recognize that the adaptation process must be done in consultation with the community. This is one area where they did not perceive significant challenges.

#### Step 6: Implementing and interpreting evidence

What and how they implement depends on the issue for which they need evidence, the type of evidence available, and how they interpret it. Interpretation is often dependent on the local context and the purpose for which the evidence will be used. Participants report regularly using evidence to support planning and implementation of PH programs and services. Our data on implementation are a bit thin but it is clear that there is strong support of the need for evidence, including research evidence, to implement relevant effective PH programs.

#### Step 7: Evaluating evidence use

Although some participants have evaluation skills or they are available in the organization, most practitioners tend to use informal versus formal evaluation processes. In this there is reliance on “if the community is happy then so are we”. There is limited support and resources in the organization to allow for evaluation.

Overall, we conclude that, in general, the individual practitioners we interviewed had a fairly good level of evidence literacy, with most people expressing some limitations in one or more aspects. At the same time, across each of the three HAs they represented, there was a high level of collective evidence literacy; high enough to provide a good foundation for building further organizational capacity for evidence literacy in public health. The areas in which there were challenges include the steps of appraisal, synthesis, and evaluation. With respect to appraisal and synthesis, there was limited if any guidance available in the EIPH framework for the kinds of appraisal and synthesis that PH practitioners saw as most important in their work. This had to do with their strong belief in the importance and value of multiple types of evidence, and the need to bring together, appraise, and synthesize these multiple forms of evidence. The challenges with respect to evaluation were most strongly related to limited organizational capacity to do evaluation.

There is an assumption that practitioners should be evidence literate, or that health authorities should be supporting the use of evidence in practice. We have been teaching research utilization and evidence-based practice for decades in Canadian health science universities, and the valuing of evidence is reflected by study participants. There can be problems with relying too much on needing evidence that programs or innovations cannot move forward without the supporting evidence. To combat this, some authors advocate for practice-based evidence, where researchers and practitioners can design and test programs that work in real-world settings [13]. This idea is congruent with statements by some of our participants and may be why they talked about the importance of evidence that is derived from community members. Additionally, Newton [37] describes how narrow the use of evidence can be when there is an expectation that knowledge should be translated in a linear process. Therefore, it is important to be balanced in the approach to evidence, recognizing the need for and limitations of using evidence in practice.

### What can we do to improve evidence literacy?

Relying on qualitative data, and with no formal test of “evidence-literacy performance”, it is possible that participants over represented their evidence-literacy. Therefore, although we believe that PH practitioners in our study are generally evidence literate, there are always opportunities for improving PH through the use of evidence. There are important questions about evidence literacy that existing frameworks do not address, such as how to appraise, synthesize and integrate locally relevant knowledge, as well as how to synthesize across multiple and different sources of evidence. Given that the EIPH framework was developed for use of research evidence, it is not always clear when and how this integration might occur within the process of evidence-informed public health.

With respect to the gap in the practice of synthesizing community-based evidence, local surveillance or big data, with research evidence, there may be existing tools that could be integrated into the EIPH framework. Kothari et al. [38] describe how the use of matrices and worksheets can assist in synthesizing local knowledge. These authors also recommend the use of deliberative dialogues, where groups help integrate and interpret local and more traditional scientific evidence. Other tools and frameworks provide more clearly for the use of multiple sources of evidence in PH practice. For example, the Program Evidence Tool “supports use of a wide range of evidence sources, including randomized studies, needs assessments, local evaluations, and stakeholder opinion” [39] (p. 7 of 9). Such a tool allows for a variety of viewpoints, and supports the use of an *evidence collection spreadsheet* to synthesize evidence.

Community generated knowledge can also be facilitated by the use of community-based or participatory approaches to research but such processes require additional time, resources, expertise and partnerships [34, 40]. In keeping with our participants’ strategies to interpret evidence in light of their PH contexts, “if reviews of intervention evidence are to be useful to decision-makers at all, contextual and implementation information is an essential, non-negotiable component of the review process” [41] (p. 462). This draws attention to the importance of newer research syntheses approaches such as realist synthesis that seeks to explore the influence of context as part of knowledge development [42]. Pettigrew [43] reinforces this position, arguing that synthesized research should rely on studies with different designs and contexts, which realist synthesis explicitly addresses.

Although not directly stated by participants, it is clear that there are important values related to, and the privileging of, certain kinds of evidence that impact the importance given to community relevant knowledge in the process of evidence informed decision making. Interestingly, in our data there are examples in which practitioners sought to challenge hierarchies of evidence that privileged traditional sources of empirical knowledge that are often seen as most valuable by researchers. Newton [37] argues, “The discourse of knowledge translation does not just require a more explicit use of certain knowledge (evidence), but instead it represents a re-organization of knowledge within the discursive field of healthcare” (pg 147). In other words, if we interpret some forms of evidence as more legitimate than others then certain forms of knowledge are downgraded. It may be more useful to think of specific sources of knowledge as important to different stages of the EIPH process such as adaptation and implementation which require local contextual and community-based knowledge.

### Do we need adaptations to the EIPH framework?

Overall, the EIPH framework is a helpful guide to developing and improving evidence informed public health practice and decision making. For the most part, however, it provides guidance in relation to the use of research-derived evidence to support practice and decision making but we believe that some modifications would be very helpful to PH practitioners. The framework privileges empirically derived research evidence, and does not provide guidance on appraising or synthesizing all of the types of evidence that practitioners actually use. This was surprising to us given the fact that the EIPH framework explicitly states that evidence-informed decision making draws on four other types of evidence in addition to research evidence. The synthesis step in the EIPH framework emphasizes the retrieval of already synthesized evidence in the form of systematic reviews and yet practitioners report needing to synthesize diverse evidence types to make decisions in practice. There is a heavy emphasis on appraising research from a bio-medical perspective, rather than appreciating the value of community-based evidence that practitioners actually rely on and trust. Where and how are practitioners developing the expertise to appraise and integrate various types of evidence?

We agree with the importance of research-based practice in PH and laud the work of the NCCMT in developing and disseminating tools and frameworks to guide evidence-informed public health practice and decision making. But, practitioners use and value multiple forms of evidence and this seems unlikely to change anytime soon. They need practical guidance in how to appraise and synthesize a range of evidence types. We strongly encourage the NCCMT to consider making adaptations to their EIPH framework to address the gaps in evidence literacy we found in this study. Given the experience PH practitioners have developed on their own and in the context of grappling with their practical evidence needs, they could be important allies and informants in the process of adapting the EIPH framework to accommodate their learning and practice needs.

### Study limitations

There are a number of limitations to this study. Because we did not set out with this evidence framework as an a priori interview guide, participants did not speak directly to each of the questions posed in the evidence framework (see Table 1), so there may be some gaps in the data. Nonetheless, implicit in their explanations of how they source, assess, apply and evaluate evidence within their work, we found answers to the questions posed in the EIPH framework through an inductive analysis of their reports of how they understood and used evidence in practice.

Strengthening evidence literacy in PH is an essential aspect of public health improvement processes. Greater gains can be made by embracing the roots of public health by valuing community-based evidence, while supporting and learning from practitioners in the work they do.

## Conclusion

This study provided an in-depth look at public health practitioners understanding and use of evidence. We found that practitioners interviewed in these two studies demonstrated a good level of evidence literacy. This was particularly evident at the collective level within the organization. While the EIPH framework provides helpful guidance in how to use research evidence, the framework lacks in providing needed support on how practitioners can appraise and synthesize across various types of evidence they consider essential in their practice. Public health practitioners use and value a range of evidence and need tools that take this range into consideration to apply all evidence to practice.

## Abbreviations

BC: British Columbia
EIPH: Evidence Informed Public Health
HAs: Health Authorities
HL: Healthy Living
KTA: Knowledge to Action
MoH: Ministry of Health
NCCMT: National collaborating Centre for Methods and Tools
PH: Public Health
